# Gold Nanoraspberries for Surface-Enhanced Raman Scattering: Synthesis, Optimization, and Characterization

**DOI:** 10.1021/acsomega.4c08791

**Published:** 2025-01-28

**Authors:** Megha Mehta, William Skinner, Benjamin Gardner, Sara Mosca, Francesca Palombo, Pavel Matousek, Nick Stone

**Affiliations:** † Department of Physics and Astronomy, University of Exeter, Exeter EX4 4QL, U.K.; ‡ Central Laser Facility, Research Complex at Harwell, STFC Rutherford Appleton Laboratory, UKRI, Harwell Campus, Oxfordshire OX11 0QX, U.K.

## Abstract

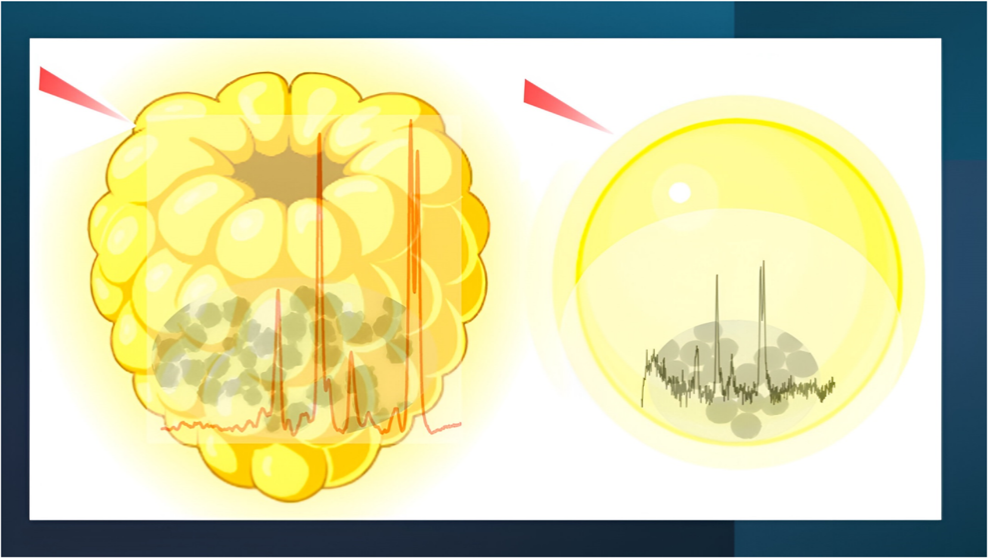

In this work, we demonstrate the synthesis of gold nanoraspberries (AuNRB) using a HEPES buffer at room temperature. The study aimed to identify and compare the physicochemical conditions of the AuNRB and gold nanospheres (AuNS) of similar size to a selected set of reporter molecules. The dispersion stability of shape-controlled and AuNS of similar diameters was investigated in three different physiological media, ultrapure water, phosphate-buffered saline (PBS), and fetal bovine serum (FBS), and compared to understand the effect of NP shape, dispersion stability, and surface-enhanced Raman scattering (SERS) enhancement. We have used two nonresonant reporters, trans-1,2-bis(4-pyridyl) ethylene (BPE) and biphenyl-4-thiol (BPT), and a resonant reporter, IR820 (also known as new indocyanine green), a clinically approved dye for diagnostic studies, to explore the relative benefit of using molecular electronic resonance, i.e., comparing SERS vs surface-enhanced resonance Raman scattering (SERRS) with these nanoparticles. SERS has been explored extensively for biomedical applications, but the synthesis of bright gold nanoparticles and the appropriate Raman label is still challenging. To understand and optimize the SERS process, we have characterized both types of gold nanoparticles, ranging from their average size, ζ-potential, and ultraviolet–visible (UV–vis) absorption. It has been found that AuNRB and AuNS are most stable when dispersed in ultrapure water, while significant aggregation of both types has been observed when dispersed in PBS. With 10% FBS, there was a slight shift and increase in the surface plasmon absorbance peak, which resulted from an increase in particle size due to protein corona formation around the gold nanoparticles. For SERS efficiency, it has been found that AuNRB outperform AuNS with all reporters. Further, the resonant reporter, IR820, has provided a higher SERS signal compared to BPE and BPT and with its FDA approval for clinical use is clearly a strong candidate for future *in vivo* application.

## Introduction

There has been a rapid increase in research reporting the use of nanotechnology for biomedical applications using novel nanoparticles.^1−4^ Nanoparticles can have unique physicochemical characteristics and provide promising prospects for developing new noninvasive methods for diagnosing and treating cancer.^5^ The optical properties of various nanoparticles are known to change with variations in size, shape, and surface charge, and as more types become available, there is a need for a new methodology for characterizing nanoparticles (NPs).^6^ Therefore, it is necessary to follow a robust protocol for the characterization of nanoparticles, to ensure that improvements in nanoparticle performance can be appropriately benchmarked in future.^7^

Gold nanoparticles (AuNPs) are generally considered biocompatible due to the relatively inert nature of the gold. Their surface chemistry and optical properties have several advantages for various biomedical applications, such as labeling, delivery, heating, sensing, and surface-enhanced Raman scattering (SERS).^8,9^ SERS is a powerful inelastic scattering technique where the Raman cross-section of molecules is enhanced by several orders of magnitude, when brought in close proximity (∼1–10 nm) to noble metal nanoparticles or roughened/nanostructured metallic surfaces. Most of the enhancement comes from the significant increase in the electromagnetic field produced by the surface plasmons in the metal, induced by the incident electromagnetic waves (light), giving rise to the phenomenon known as SERS.^10,11^

It is well known that controlling the shape of gold nanostructures enables one to specifically modify the physical and chemical properties for a range of promising biomedical applications.^12^ Several studies have reported the synthesis of AuNPs with anisotropic features, such as nanorods,^13−15^ nanostars,^16−18^ dendritic,^19−21^ flower-like,^22−24^ and multibranched^25,26^ nanostructures, and demonstrated excellent potential for biomedical applications in chemical sensing using SERS.^27^ Such anisotropic AuNPs are of particular interest because minor modifications in their shape can enable them to generate SERS “hot spots”, locations whereby molecules can be exposed to higher electric fields and thus dramatically improve the signals obtained and the sensitivity of SERS. Care needs to be taken when synthesizing, functionalizing, and characterizing more complex nanoparticles, especially when they need to be exposed to the biological milieu.

A conventional method of preparing gold nanoparticles is the simple reduction of metal salt precursors with reducing agents under controlled conditions, in either water or organic solvents, and attaching the thiol ligands to stabilize the nanoparticles by controlled binding to the surfaces of the particle.^28^ Herein, we present a simple one-pot method to synthesize AuNRB using a “green” chemical, 2-[4-(2-hydroxyethyl)-1-piperazinyl]ethanesulfonic acid (HEPES) buffer, also known as Good’s buffer extensively used in chemistry and biochemistry laboratories because its p*K*_a_ is close to physiological pH. HEPES buffer acts as a reducing and shape-directing agent, therefore used for the synthesis of gold nanoparticles.^29−32^ The current research work is the first demonstration of synthesizing aspherical gold nanoparticles by tuning the HEPES buffer and gold concentration to obtain raspberry-like nanostructures, that we call gold nanoraspberries, and due to the nanostructured surface undulations, we found it to be a significantly better enhancer than the commonly used and similar sized AuNS.

The study was systematically conducted in two steps.

First, to identify and compare the physicochemical reaction parameters of AuNS and AuNRB in different physiological media that can provide valuable information about the nature of nanoparticles and the best conditions to avoid aggregation, it is critical to characterize the nanoparticles in a relevant medium for their planned deployment and to understand the effect of aggregation, destabilization, or dissolution of nanoparticles, which may otherwise lead to erroneous interpretation of results.

The second is to compare the SERS efficiency of AuNRB and AuNS in selected media using Raman reporter molecules that have strong Raman scattering signals, with “sharp peaks” which provide a unique SERS NP “flavor” and allow for high sensitivity and specificity. Nonresonant SERS reporters have been most commonly used, but more recently, resonant labels producing surface-enhanced resonance Raman spectroscopy (SERRS)^33−35^ have gained popularity. These labels or dyes have a molecular electronic transition in resonance with the excitation wavelength, thus generating additional enhancement of the Raman signal as compared to SERS, although this can be accompanied by a fluorescent background that is not always quenched by being in the vicinity of the nanoparticle.

For our study, we have synthesized AuNRB of 59 ± 5 nm diameter and benchmarked them against commercially available AuNS of similar size 60 ± 5 nm (NanoXact, NanoComposix, AUCN60-NCX). For stability tests, we have used three physiological media, ultrapure water, 1× phosphate-buffered saline (PBS), and 10% fetal bovine serum (FBS). Later, to compare AuNRB and AuNS dispersed in selected media for SERS efficiency, we have used two nonresonant reporters—trans-1,2-bis(4-pyridyl)ethylene (BPE) and biphenyl-4-thiol (BPT), and a resonant reporter, IR 820 (also known as new indocyanine green), a clinically approved dye for diagnostic studies,^36,37^ to explore the relative benefit of using molecular electronic resonance, i.e., comparing SERS vs SERRS with these NPs.

NP characterization was conducted by using UV–visible absorption spectroscopy to provide a measure of the localized surface plasmon resonance (LSPR). Dynamic light scattering (DLS) was used to measure the hydrodynamic particle size by observing the time-independent fluctuations in the intensity of scattered light caused by particle motion, which can be directly correlated to the particle’s diffusion coefficient in the dilute sample.^38,39^ Transmission electron microscopy (TEM) was employed to measure the diameter of electron-dense nanoparticles and to resolve structures at an angstrom level.^40^ Finally, Raman spectroscopy measurements were performed to identify the specific biomarkers for SERS^41^ to provide the best combination of ‘bright nanoparticle–dispersing media–reporter’, which further facilitate SERS and SERRS and their applicability for biological and clinical applications.

## Experimental Section

### Chemicals and Reagents

Gold(III) chloride hydrate (HAuCl_4_·*x*H_2_O, 99.995%), 2-[4-(2-hydroxyethyl)- 1-piperazyl] ethanesulfonic acid (HEPES), sodium hydroxide (NaOH), and phosphate-buffered saline (PBS) were purchased from Sigma-Aldrich (St Louis, MO). Spherical gold nanospheres, 60 nm in diameter (NanoXact) at 0.05 mg/mL in aqueous 2 mM sodium citrate, were purchased from nanoComposix. Reporters 1,2-bis(4-pyridyl)ethylene (BPE, 98%), biphenyl-4-thiol (BPT), and IR 820 (2-[2-[2-chloro-3-[[1,3-dihydro-1,1-dimethyl-3-(4-sulfobutyl)2H-benzo[*e*]indol-2-ylidene]-ethylidene]-1-cyclohexen-1-yl]ethenyl]-1,1) were purchased from Sigma-Aldrich (St Louis, MO). Ultrapure water of HPLC grade and Gibco fetal bovine serum (FBS) were ordered from ThermoFisher Scientific Inc.

### Synthesis of Multibranched Gold Nanoparticles and SERS Tag

A 20 mM HEPES solution was prepared by adding 2 mL of 100 mM HEPES to 8 mL of ultrapure water.^29,30^ The pH of the solution was adjusted to 7.4 by adding 1 M NaOH. Then, 0.25 mL of 20 mM HAuCl4 was added, and the colorless solution turned turbid blue within 30 to 45 min. The solution was left overnight at room temperature, and the next day, it was centrifuged at 5500 rpm for 15 min and the pellet was resuspended in ultrapure water. To investigate the best dispersion media for resuspending the obtained nanoparticles, we have prepared matching samples each with 1 mL of AuNRB suspension, centrifuged at 5500 rpm for 15 min, and redispersed in either ultrapure water, PBS, or 10% FBS. Similarly, we prepared three samples of commercially purchased 60 nm nanoComposix spherical gold nanoparticles by centrifuging and then redispersing in ultrapure water, PBS, or 10% FBS. Both AuNRB and AuNS were characterized using UV–visible spectroscopy, DLS, and TEM to understand the dispersion status of each nanoparticle solution.

For SERS characterization, we have used three labels, two nonresonant, BPT and BPE, and one resonant label, IR 820, to understand the SERS signal enhancement of these labels with different types of nanoparticles. We have added 100 μL of 5 μM BPT or BPE or 70 μL of 5 μM IR 820 to 900 μL and 930 μL of nanoparticle suspensions to get a final volume of 1 mL and a reporter molecule concentration of 5 × 10^–7^ M. The suspensions were vortexed (Vortex Varimix shaker (SciQuip)) for 1 min, incubated for 2 h at room temperature, and centrifuged at 4500 rpm for 15 min. The supernatant was discarded, and the pellet was redispersed in water. The centrifugation process was repeated twice to remove any residual-free label, and the final obtained pellet was redispersed in ultrapure water, PBS, or 10% FBS to obtain a final volume of 1 mL. The final labeled nanoparticle solution was used for SERS and other characterization measurements such as UV–vis, DLS, TEM, and Raman scattering.

### Instrumentation

#### UV–Visible Spectroscopy

UV–visible spectra were acquired using an Evolution Array UV–visible spectrophotometer in the range of 400–1100 nm with a 1 cm-path length cuvette. The wavelength of maximum absorbance and shape of the localized surface plasmon resonance (LSPR) were analyzed by using OriginPro 2023b software.

#### Dynamic Light Scattering

The hydrodynamic size of AuNS and AuNRB before and after labeling with reporter molecules in different media was determined by dynamic light scattering (DLS) using Malvern Zetasizer Ultra running DTS software and a 4 mW He–Ne laser at 633 nm. Analysis was performed at an angle of 90° and a constant temperature of 25 °C. Disposable cuvettes of 1 cm path length were employed for size measurements, whereas surface charge ζ-potential measurements were performed in a capillary folded disposable cuvette. Each value was the average of three independent measurements.

#### Transmission Electron Microscopy

A JEOL 2100 TEM instrument was used to study the NP morphologies on 200-mesh holey C-coated copper grids at 100 kV. The as-prepared hybrid samples were diluted 100-fold and deposited on the TEM grids to minimize any drying artifacts. The sizes were determined using ImageJ software by measuring ≈60–70 individual assemblies per sample.

#### Raman Spectroscopy

SERS measurements were carried out using a hand-held CBEx spectrometer (Snowy Range), with 785 nm laser excitation wavelength. All measurements were performed in triplicates using a laser power of 12 mW at the sample and 1 s integration time, with five accumulations, in the spectral range of 400–2300 cm^–1^. Low-volume glass vials, for use in the instrument sample compartment, were used as sample holders, and all SERS spectra were obtained from suspended colloidal samples. Each spectrum was preprocessed with an algorithm written using the SciKit Learn package^23^ in Python 3.7. Baseline correction using asymmetric least-squares algorithm, background subtraction, and average spectra of triplicate measurements were performed using the Python code written in Jupyter notebook using Anaconda Navigator. Then, spectral normalization was conducted by dividing the whole spectrum by the gold concentration of each type of nanoparticle using Origin 2023b (Origin Lab Corporation, Northampton, MA).

## Results and Discussion

**Scheme 1:**
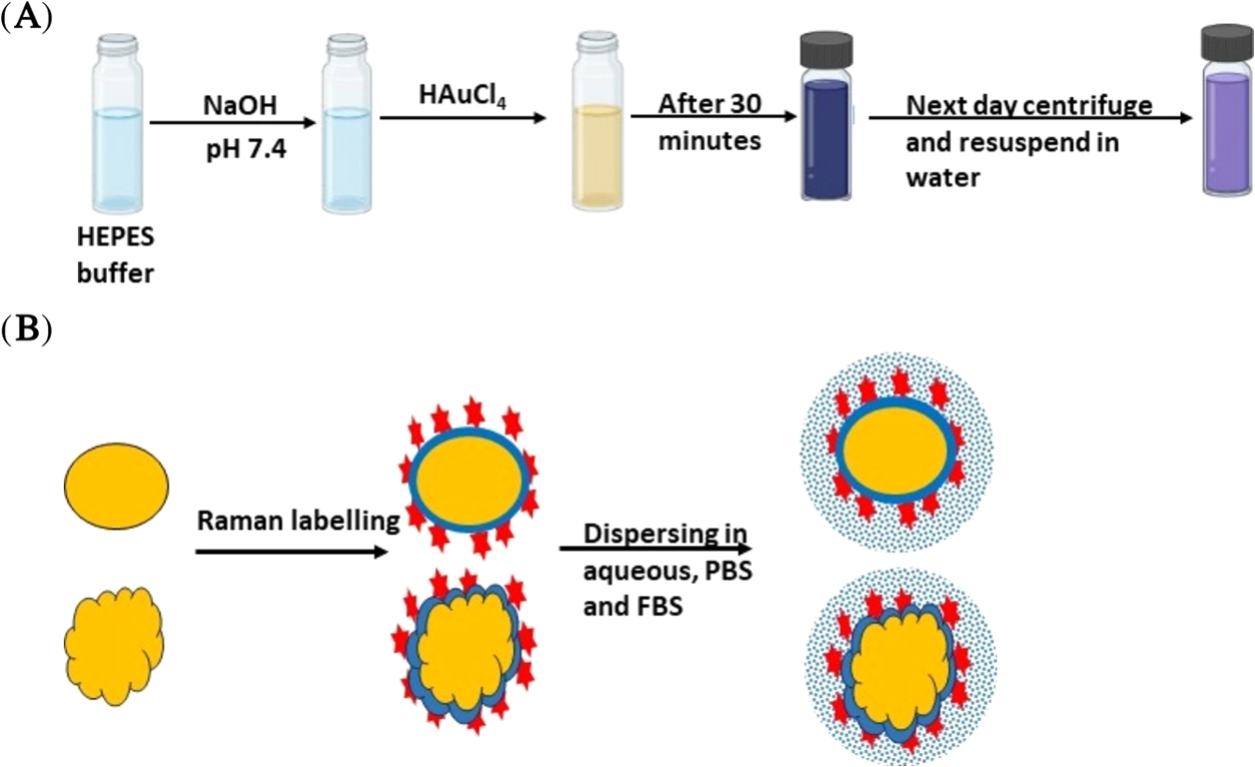
(A) Schematic Illustration of Preparation of AuNRB and (B) Labeling with Raman Reporters Followed by Resuspension in Media

A schematic diagram showing the synthesis of AuNRB and their labeling with Raman reporters is shown in Scheme 1.

For the preparation of AuNRB, we have used HEPES buffer also known as Good’s buffer, which has piperazine groups that produce nitrogen-centered free radicals that reduce the trivalent gold ions to divalent gold ions and monovalent gold ions and finally into gold zerovalent ions, which is due to the aggregation of the reduced gold ions. This reaction proceeds until the availability of gold ions is ended, and after that, the HEPES acts as a stabilizing agent.^29−31^ In this study and elsewhere, it has been shown that the shape of gold nanoparticles plays a dominant role in determining the surface enhancement. The gold nanoparticles used in this study were characterized by the combination of UV–visible absorption spectroscopy, TEM, DLS, and SERS, and the results obtained are presented in Table 1.

**Table 1:** Physical Parameters of the Gold Nanoparticles Used in This Study

types of nanoparticles	hydrodynamic diameter (nm)	particle concentration (p/mL)	ζ-potential (mV)	absorbance maximum (nm)
AuNRB	59 ± 5	2.65 × 10^10^	–41	569
AuNS	60 ± 5	2.04 × 10^10^	–46	532

### UV–Visible Spectrophotometry

To identify whether the particles are “dispersed” or “aggregated”, we have carefully examined the stability of AuNRB and AuNS in different dispersing media: aqueous, PBS, and 10% FBS. The nanoparticles’ surface plasmon resonance depends on the strong absorption of electromagnetic waves in the visible range, which causes collective oscillations of the conduction band electrons of the gold.^42^ This can be confirmed by UV–visible measurements, which determine the absorbance (really extinction but the contribution of scattering is ignored in these measurements) of the nanoparticles. In general, it has been reported that the absorbance maximum shifts to longer wavelengths with increasing particle size.^12,43^

**Figure 1:**
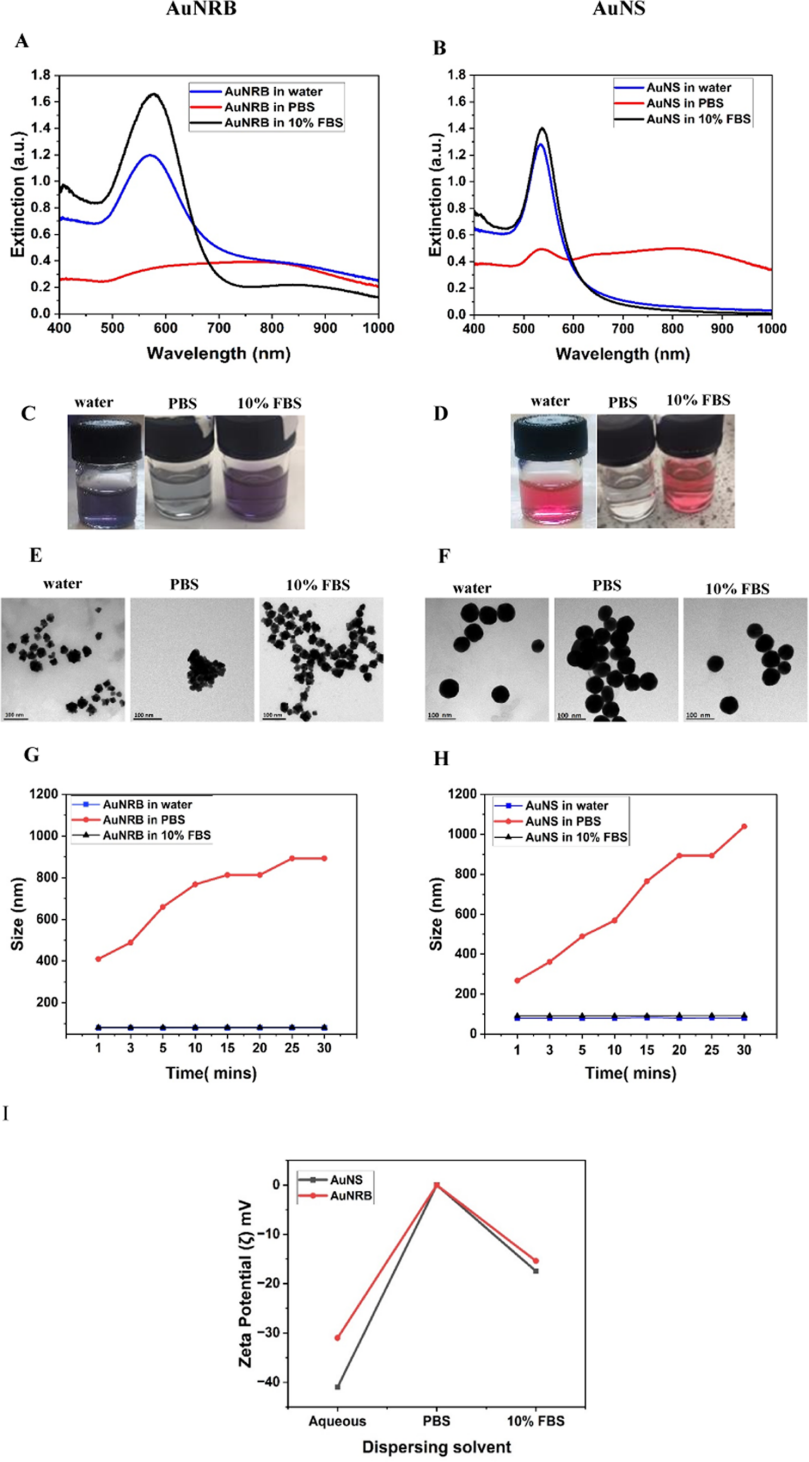
(A, B) UV–visible spectra; (C, D) photographic images; (E, F) TEM images; (G, H) DLS size measurements at different times; and (I) ζ-potential of AuNRB and AuNS dispersed in water, PBS, and 10% FBS.

Figure 1A,B shows the UV–visible spectra of AuNRB and AuNS in aqueous, PBS, and 10× FBS suspensions. The spectral profile of AuNRB in an aqueous suspension shows that the surface plasmon resonance (SPR) maximum absorbance peak at 569 nm with the shoulder peak at 800 nm is observed, which is indicative of chain plasmon modes formed in aggregates. This second LSPR shows the synthesis of AuNRB of size around 59 nm agglomerate on the surface of an aspherical gold metal core to evolve into diverse shapes and structures, which results in the desired morphology. In many cases, it appears as a shoulder rather than an isolated peak, depending on the degree and morphology of the aggregation. We have added NaOH to adjust the pH of HEPES solution to 7.4, which stabilizes the AuNRB solution for long-term use. Surface plasmon resonance (SPR) of AuNS shows the maximum absorbance at 532 nm when dispersed in water. Both AuNRB and AuNS when dispersed in PBS dramatically reduced their maximum SPR band and a second broad SPR peak was apparent in the near-infrared region, which is caused by aggregation of the nanoparticles.

When looking at the vial, the discoloration of nanoparticles after the addition of PBS can be clearly seen, as shown in Figure 1C,D. To improve the dispersion of the selected gold nanoparticles and to understand the intrinsic complexity of the biological milieu, fetal bovine serum (FBS) was explored. Dispersing the AuNRB and AuNS in 10% FBS results in a higher absorbance of 1.6 and 1.4 with a slight red shift of the SPR band from 532 to 540 nm because of increasing particle size with the formation of agglomerates. Based on most of the reported protocols found in the literature,^44−46^ nanoparticles suspended in 10% FBS remain colloidally stable with a slight red shift of the LSPR band due to the occurrence of a protein corona on the nanoparticle surface. FBS has been shown to stabilize NPs compared to NPs dispersed in an aqueous solution.

### Transmission Electron Microscopy

Transmission electron micrographs were obtained from AuNRB and AuNS dispersed in the selected media to evaluate the precise size and degree of aggregation in accordance with the UV–visible and DLS data (Figure 1E,F). The mean size of AuNS in aqueous solution measured using ImageJ software was 65 ± 7 nm, which matches the information provided by the supplier. There is no discernible change in the size when dispersed in FBS. This demonstrates that the particles are monodisperse and free of aggregation. Similar behavior was observed when AuNRB were dispersed in aqueous media and FBS with particle sizes of around 60 ± 7 nm. TEM pictures of AuNRB in various dispersing media at varying magnifications are mentioned in Supporting Information Figure S2 with a representative scanning electron microscope image of AuNRB in aqueous media (Figure S3). Both types of gold nanoparticles showed aggregation and cluster formation in accordance with the increase in hydrodynamic diameter and ζ-potential when dispersed in PBS as found from DLS measurements, resulting in a decreased repulsion between the NPs and a shift of absorbance to a higher wavenumber.

### Dynamic Light Scattering

Dynamic light scattering (DLS) is the most commonly used method to measure the size and polydispersity of suspended particles in a sample.^47,38^ DLS measurements of the AuNS indicate monodisperse nanoparticles with a hydrodynamic diameter of 60 ± 5 nm and a ζ-potential of −46 ± 3 mV. The particle surface of commercial AuNS was citrate-capped with a mass concentration of 0.053 mg/mL. The in-house synthesized AuNRB shows a monodisperse suspension with a hydrodynamic diameter of 59 ± 5 nm and a ζ-potential of −40 ± 3 mV. The AuNRB and AuNS dispersed in PBS suffered a rapid rise in particle size due to aggregation and then crashing out of solution (when the mass exceeds the ability of the solvent to maintain the suspension), and more than 90% of suspensions suddenly became colorless in the first 30 min. This could be due to the PBS salt solution, which reduces the electrostatic stabilization of the gold nanoparticles resulting in rapid “crash”, that is, the gold nanoparticles aggregate and precipitate. In contrast, in water and 10% FBS, the dispersions were observed to be stable and highly monodispersed. The results are shown in Figure 1G,H.

The ζ-potential value of AuNS in ultrapure water was found to be −44 ± 1.5 mV (Table 1). A slight decrease in ζ-potential to −15 ± 2 mV was observed when AuNS were dispersed in 10% FBS and became nearly neutral with 1× PBS (Figure 1I).

Similarly, the ζ-potential of AuNRB in ultrapure water was found to be −41 ± 3 mV (Table 1). A slight decrease in ζ-potential to −15 ± 1 mV was observed when AuNRB were dispersed in 10% FBS and became nearly neutral with 1× PBS. The sudden decrease of ζ-potential and absorbance to near-neutral with PBS might be due to the high ionic strength of PBS disrupting the surface charge of AuNP.^48,49^ The significant reduction in ζ-potential values of AuNP in FBS media is caused by a high ionic environment in the media, which contains a percentage of inorganic salts, amino acids, and proteins.^12,50,51^

### Localized Surface Plasmon Resonance and Molecular Electronic Resonance

In the second part of the study, we investigated the role of LSPR and the molecular electronic resonance of reporter molecules for AuNS and AuNRB when dispersed in the selected media. Specifically, to understand the plasmonic interactions, we have employed nonresonant reporters, BPE and BPT (for SERS), and a resonant dye, IR 820, for surface-enhanced resonance Raman scattering (SERRS). All of the selected reporters have a unique molecular fingerprint and a potential for bioimaging applications. These reporter molecules also have relatively high Raman cross-sections and distinguishable spectral peaks, as previously reported.^34−55^

**Figure 2:**
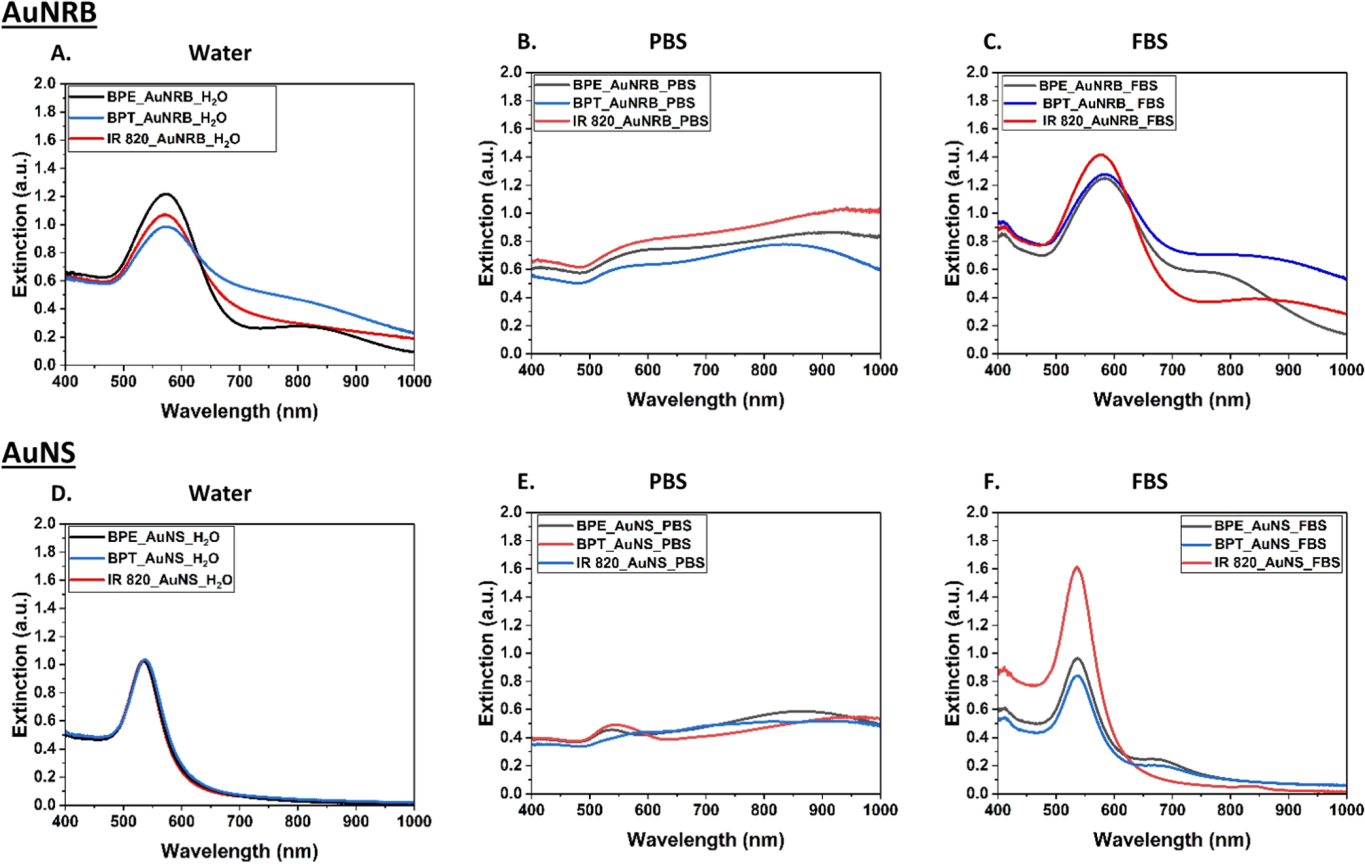
UV–visible absorption spectra showing the stability of AuNRB and AuNS after labeling with BPE, BPT, and IR 820 and dispersed in aqueous (A, D), PBS (B, E), and 10% FBS media (C, F). Samples dispersed in PBS show significant aggregation and can be seen with another broad peak in the region 750–950 nm and a decrease in the extinction.

**Figure 3:**
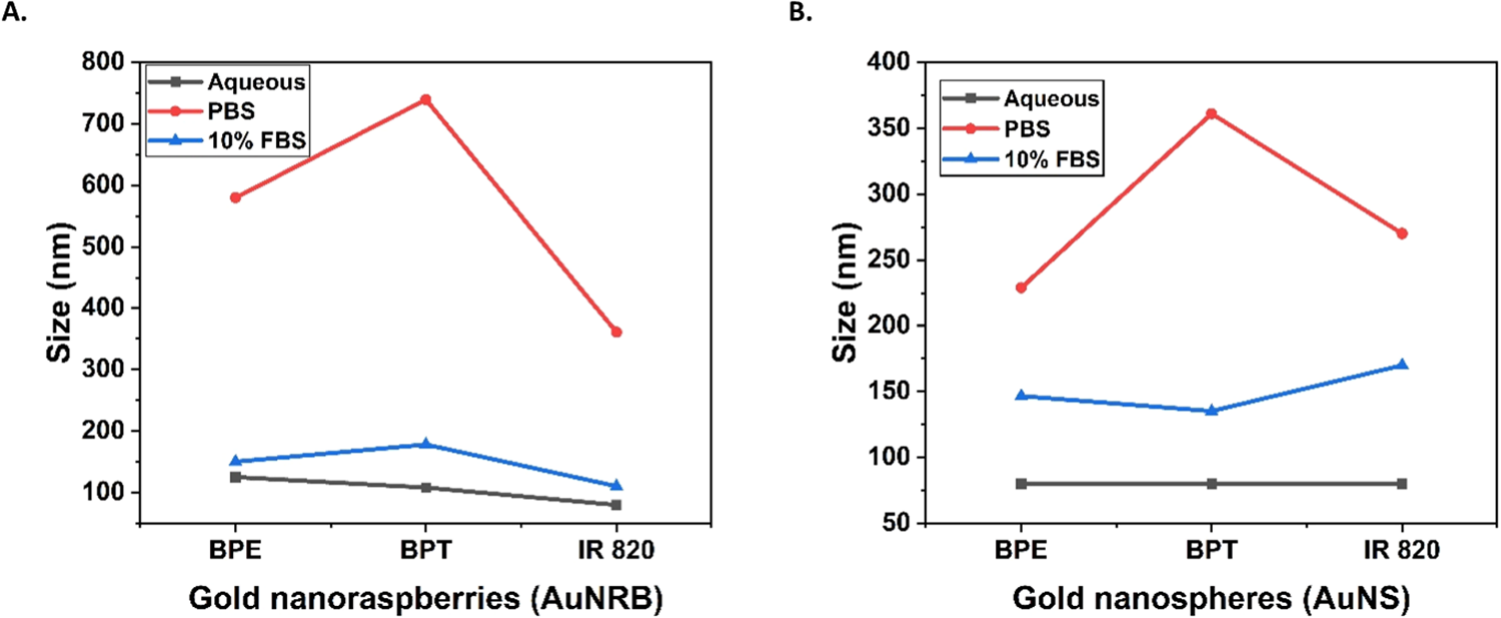
Dynamic light scattering (DLS) measurements showed the average size increased to the maximum for AuNRB (A) and AuNS (B) with all reporter molecules, BPE, BPT, and IR 820 in PBS than dispersed in aqueous and 10% FBS.

The labeled AuNS and AuNRB were characterized in a similar way using UV–visible spectroscopy, DLS, and TEM but now evaluated for SERS activity to understand the contribution from the chemical effect due to the stronger binding of each adsorbing reporter molecule to the surface of the gold nanoparticles. The plasmonic absorption band depends on the size and shape as well as the aggregation behavior of the nanoparticles. It is also known that the plasmon–plasmon interaction among the aggregated gold nanoparticles can produce a red shift in the absorption band, which is generally much broader than the individual NP plasmon resonance peak. In many cases, it appears as a shoulder rather than an isolated peak, depending on the degree and morphology of the aggregation. Also, the morphology of gold nanoparticles determines the changes in their surface environment after the adsorption of molecules of interest to the nanoparticle surface. The shift in the absorbance peak is proportional to the thickness of the adsorbed layer.^52,53^ The formation of another plasmon peak between 650 and 700 nm indicates the formation of clusters. At the same time, spherical aggregates, as formed with AuNS, show an increase in particle size with a smaller or no shift to a higher wavelength. It has been found from the UV–visible (Figure 2) and DLS measurements (Figure 3) that AuNRB behave differently from AuNS. Stability of AuNRB and AuNS after tagging with Raman reporters was also monitored at different time intervals presented in Supporting Information Figure S1. UV–visible absorbance of AuNS labeled with BPE, BPT, or IR 820 in water shows no change in the absorbance, which implies no aggregation of nanoparticles, and DLS measurements showed no change in the size of AuNS after labeling with reporters. In contrast, BPE-tagged AuNRB in water show an increase in absorbance with the second broad peak rising around 820 nm, which could be the result of the change in particle size with some aggregation of nanoparticles. This is supported by DLS size measurements (Figure 3), showing an increase in size to 125 nm due to binding to the nanoraspberry surface. This could also be due to the two pyridyl moieties of BPE that can bind with AuNP to form aggregates as dimers or trimers, but no further change in size was found when investigated for 30 min. BPT-tagged AuNRB in water also show a broad band above 700 nm, indicating the presence of small aggregates. This could be because aryl thiols form densely packed monolayers on AuNRB due to π-stacking^54^ and have an influence on the degree of aggregation, whereas the resonant dye IR 820 appears to slightly aggregate the nanoparticles without dramatically affecting the size as observed from the UV–visible spectra (Figure 2). These results demonstrate that IR 820 is the most stable reporter with both types of nanoparticles when it is dispersed in water.

**Figure 4:**
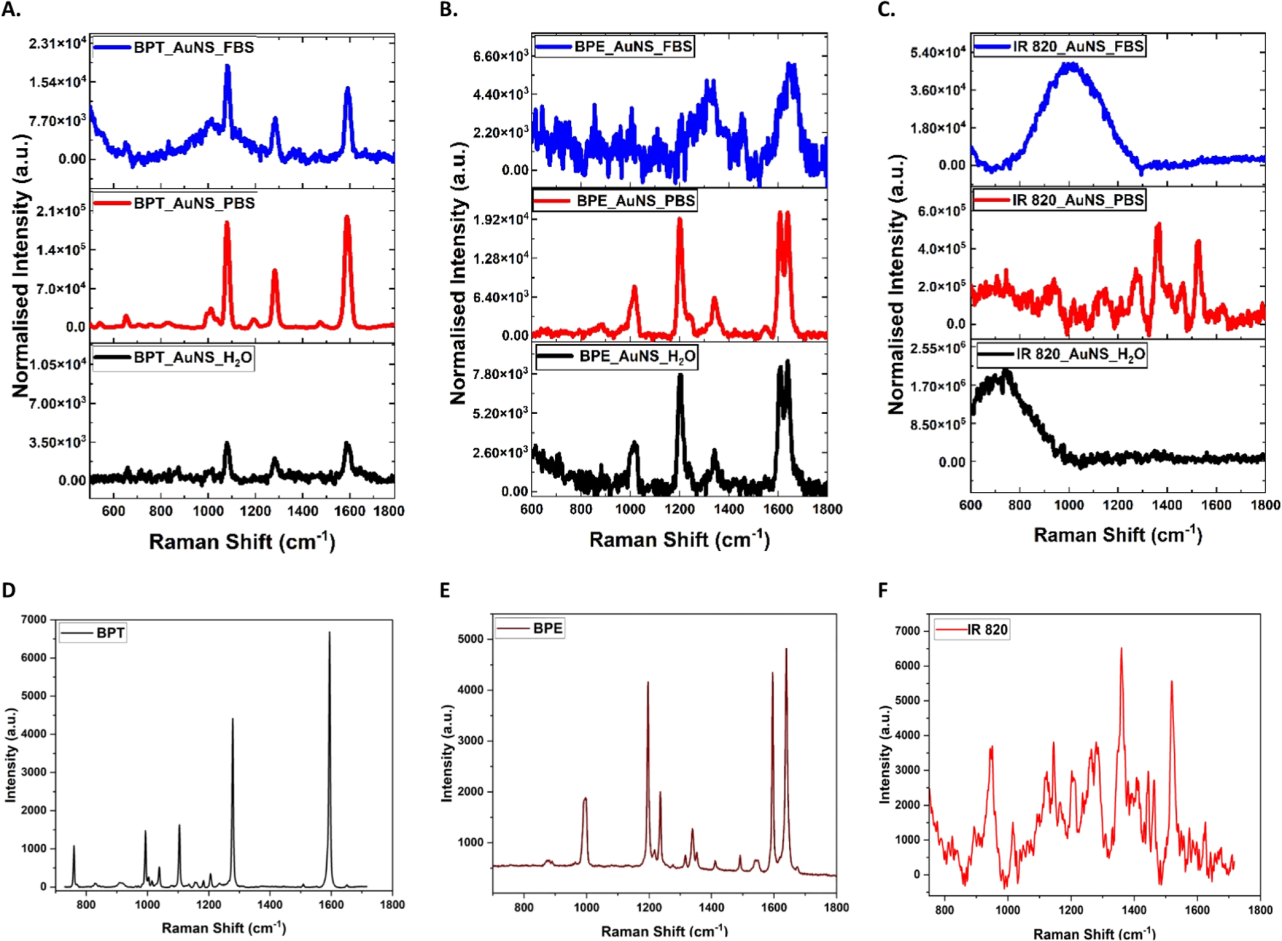
SERS and SERRS spectra of AuNS labeled with 5 μM BPT(A), BPE(B), and IR 820(C) dispersed in 10% FBS (blue), PBS (red), and water (black). (D–F) Raw Raman spectra of 1 mM BPE, BPT, and 100 μM IR 820 (IR 820 spectrum is background-subtracted to remove the fluorescence signal) reporters in bulk solution. All measurements were performed in triplicates using a laser power of 12 mW at the sample and 1 s integration time, with five accumulations using 785 nm laser excitation wavelength. All spectra have been baseline-corrected and normalized, and average spectra are presented.

The dispersion of AuNS in 10% FBS allows the citrate to be displaced on the AuNP surface, which may trigger some level of aggregation with a small shift in the absorbance peak of 4–5 nm, indicating the binding of biomolecules to the gold surface by changing the electron field. There is another surface plasmon resonance peak around 680–700 nm, which confirms the formation of small aggregates. IR 820-tagged AuNS in an FBS medium show higher absorbance compared to BPE- and BPT-tagged AuNS. This could be attributed to a feature of IR 820 molecules that tend to adsorb on serum proteins and form protein-sized “nanoparticles” and prevent IR 820 molecules, forming nanoparticle aggregates together with the gold,^37^ with AuNS and providing no surface enhancement, resulting in a very weak SERRS signal, as presented in the next section (Figure 4C).

### Surface-Enhanced Raman Scattering

The relative strength of the measured SERS labels’ signals helps us understand how the reporter molecules interact with the gold and if they are adsorbed on the surface of gold nanoparticles, providing different enhancements of SERS signals.^34^

To provide conditions close to the potential applications of plasmonic tags, an SERS study was performed with 785 nm excitation. This corresponds to a range of widely used NIR lasers for biomedical applications of Raman due to the relatively low fluorescence backgrounds induced and the absence of substantial photodamage (due to very few molecules having electronic excitation bands at NIR energies). All reporter-labeled AuNS and AuNRB in PBS show a high SERS signal, which appears to be due to the rapid aggregation of nanoparticles, forming clusters with poor stability, as demonstrated in scattering and optical characterization results. Nanotag solutions were used for all of the SERS measurements. To assess the SERS signal for each label, we identified the major peaks and their assignments. BPT has three main peaks at 1081, 1284, and 1588 cm^–1^, which correspond to a C–H rocking and two in-plane stretches of the benzene rings, respectively.^56^ BPE presents the strongest peaks at 1608 and 1636 cm^–1^ attributed to benzene stretching and the C=C stretching mode, respectively. For BPE bound through the pyridyl group, the band at 1202 cm^–1^ arises due to the aromatic ring C–C/N stretching and symmetric ring breathing mode at 1000 cm^–1^. This indicates their binding to the AuNP surface and supports chemisorption through with two chemically active nitrogen atoms in a highly conjugated structure.^34,55^ Resonant dye IR 820 shows a peak at 1523 cm^–1^, which corresponds to the pyrrole stretching, 1627 cm^–1^ due to the superposition of in-plane benzene and pyrrole stretching mode, 1446 cm^–1^ which corresponds to −NH deformation and benzene deformation, 1360 cm^–1^ due to the C–N bond stretching of the pyrrole ring, 1169 cm^–1^ for the −CH bond of the benzene ring, and 1228 and 1264 cm^–1^ for the C–C bonds from the backbone structure of polymethine. Thus, the fingerprint of the IR 820 was obtained by the SERRS effect, clearly showing the backbone, polymethine structure, indole, and benzene rings.^37,54,55^

Considering AuNS with labels dispersed in different media, it has been found that AuNS tagged with BPE, BPT, and IR 820 have strong SERS signals when dispersed in PBS due to aggregation generating more hotspots between nanoparticle surfaces in close proximity to each other ∼1–2 nm (Figure 4). AuNS tagged with BPT when dispersed in 10% FBS have a higher SERS enhancement but with a more significant background signal, which might be due to the protein corona formed by FBS on the nanoparticle hindering the binding effect. AuNS labeled with BPE have a higher surface enhancement when dispersed in water than FBS, and again, some background noise due to changes in the surface of the nanoparticles due to proteins and other small molecules affecting the SERS peaks. AuNS labeled with IR 820 have no discernible SERS signal when dispersed in water and 10% FBS. This could be due to the surface charge of nanospheres and the chemical nature of the dye in water and that 10% FBS does not induce any aggregation of the nanoparticles.

It is only when the AuNS gets highly aggregated with PBS and generates local hotspots where the local field is substantially enhanced that IR 820 can provide some SERS enhancement. There could be another reason as AuNS have an absorbance maximum at 532 nm, which is quite far from the dye excitation wavelength in the NIR region, and therefore, there is less fluorescence quenching and surface enhancement at 785 nm, resulting in a relatively weak SERS signal. Based on these SERS results for AuNS, it can be concluded that the order of performance of these reporters (at 5 μM) from the best to the worst was as follows: IR 820_AuNS_PBS > BPT_AuNS_FBS > BPT_AuNS_PBS > BPE_AuNS_PBS.

The results indicate that AuNS aggregation is the driving force behind producing the appropriate electromagnetic hotspots for maximal SERS. BPT binding with gold using a thiol moiety generates strong a SERS signal in PBS, which can be confirmed from the UV–vis and DLS measurements that after dispersing in PBS, the ζ-potential becomes neutral, and the size of particles increased tremendously, leading to the crashing out of particles.

**Figure 5:**
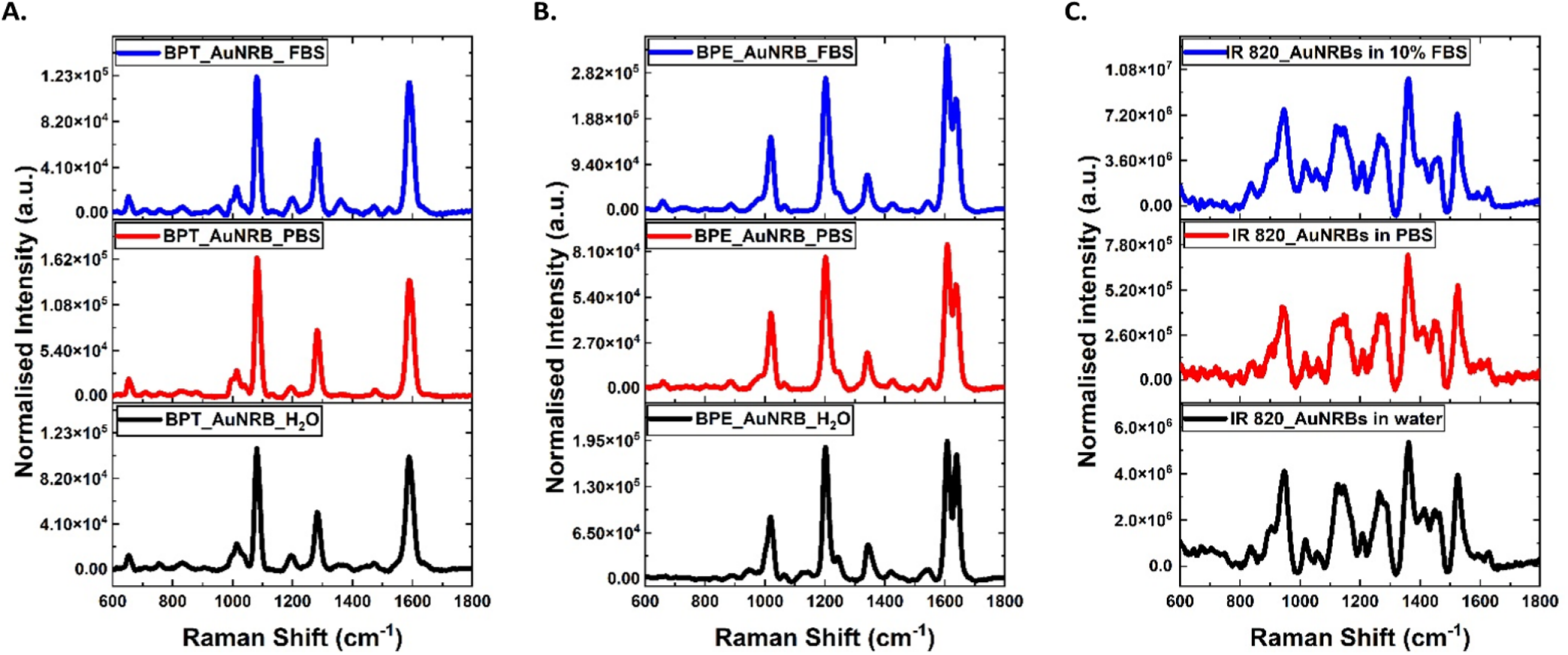
SERS and SERRS spectra of AuNRB labeled with 5 μM BPT (A), BPE (B), and IR 820 (C) dispersed in 10% FBS (blue), PBS (red), and water (black). All measurements were performed in triplicates using a laser power of 12 mW at the sample, 1 s integration time, and five accumulations using 785 nm laser excitation wavelength.

For AuNRB, the irregular surface topology gives rise to more electromagnetic hotspots, which can be accessed by the reporter labels, resulting in a strong enhancement of the SERS signal. IR 820 is a resonant dye and presents a strong absorption band at 690 nm with another shoulder band at 819 nm (UV–vis absorbance spectra of IR 820 added in Supporting Information Figure S4), which when attached to AuNS in the aggregated state generate some SERRS for AuNS whose SPR is too far from the dye excitation wavelength, whereas with the AuNRB second broad shoulder peak around 800 nm that significantly enhances the SERS signals and allows the AuNRB to be detected with all reporters and in different dispersing media because its maximum absorption point is near the excitation wavelength (785 nm) of the Raman system. The AuNRB provides a strong enhancement with BPE, BPT, and IR 820 in all dispersing media (Figure 5). Therefore, as expected, the results of this study show clearly that the morphology of the nanoparticles plays a vital role in the SERS effect and can provide the strongest enhancement with resonant labels.

Depending on the SERS performance, we evaluated SERS results to calculate the SERS enhancement factor. For all three reporters, the Raman spectra (Figure 4D–F) were obtained using 785 nm excitation wavelength. Enhancement factor, normally defined as EF, = *I*_SERS_*C*_Raman_/*I*_Raman_*C*_SERS_, where *I*_SERS_ and *I*_Raman_ are, respectively, the SERS and normal Raman intensities of the reporter molecule and *C*_SERS_ and *C*_Raman_ are the SERS and normal Raman concentration of the reporter molecule (Supporting Information 1). The SERS enhancement factor of BPT- and BPE-labeled AuNRB dispersed in aqueous was calculated to be 3.11 × 10^4^ and 8.63 × 10^4^, respectively. The Raman mode of BPT at 1588 cm^–1^, BPE at 1608 cm^–1^, and IR 820 at 1360 cm^–1^ was chosen for the EF calculations. For the resonant molecule, IR 820, the SERRS enhancement factor with AuNRB was calculated to be 1.64 × 10^6^. The SERS reporter concentrations were assumed to be the maximum reporters available, i.e., the resultant enhancement factor is likely to be higher as all molecules will not have adsorbed to the surface with some washed off.

From the obtained results, it is evident that most of the SERS enhancement obtained from gold nanoparticle suspensions comes from some form of aggregation. However, AuNRB tagged with the resonant label IR 820 provide strong SERRS with very little aggregation and appear to provide 100 times more signal from hotspots than SERS signals (Figure 5) obtained from BPE- and BPT-labeled AuNRB. This study demonstrates how anisotropic AuNRB with the right selection of dispersing media and Raman reporter can achieve strong SERS/SERRS signals, even if no signal can be obtained with spherical gold nanoparticles of similar size. Based on these SERS results from AuNRB, it can be concluded that the order of performance of these reporters (at 5 μM) from the best to worst was as follows: IR 820_AuNRB_FBS > BPE_AuNRB_FBS > BPT_AuNS_FBS. The results indicate that AuNRB shows stronger SERS signals with reporter molecules when dispersed in FBS than with PBS and water, which makes these nanoparticles a good candidate for diagnostic applications in different biological media for future studies.

It has been found that using the right Raman reporter and nanoparticle size can increase the Raman scattering cross-section by several orders of magnitude. The experimental results are explained by considering the molecular cross-section for resonant Raman scattering and the local electromagnetic enhancement factor at the surface of the gold nanoparticles. These findings may help the selection of appropriate gold nanoparticle–SERS label combinations for improved performance.

## Conclusions

The results of this study demonstrate that the AuNRB are the most effective choice of NP (versus nanospheres) when using the specific selection of reporter molecules and media tested. Overall, the anisotropic AuNRB outperforms the AuNS in terms of the SERS signal with all selected reporters, BPE, BPT, and IR 820, when dispersed in aqueous media compared with other selected media. Ultrapure water is considered a suitable medium for both types of gold nanoparticles with no significant aggregation and suspension of nanoparticles, as opposed to what is observed with PBS. The resonant reporter IR 820 with AuNRB has shown a strong SERS enhancement with an enhancement factor of 1.64 × 10^6^ and as a clinically approved contrast agent can be considered a strong candidate for future work on in vivo multiplexed disease detection.

Since biological applications require stable suspensions of gold nanoparticles, AuNRB with labels dispersed in water are the most stable with a higher SERS signal, although this would change as soon as the NPs enter the biological milieu. These differences may be related to different molecular Raman cross-sections, variations of Raman reporters’ density at the surface of the NPs due to different binding affinities, and the varying stability in different dispersion media. Understanding such physicochemical characteristics of nanoparticles in conjunction with the right dispersing environment and reporter will help in translating nanomedicines from the benchtop to the clinic.

## Supporting Information Available

DLS size measurements of AuNRB and AuNS tagged with 5 μM BPE, BPT, and IR 820 and later dispersed in ultrapure water, PBS, and 10% FBS at different time intervals to determine the stability of nanoclusters; TEM images of different magnifications of AuNRB dispersed in water, PBS, and 10% FBS; SEM images of AuNRB dispersed in water; and UV–vis absorbance spectrum of 5 μM IR 820 in water and enhancement factor calculation (PDF)

## Abbreviations

SERS: surface-enhanced Raman scattering
SERRS: surface-enhanced resonance Raman scattering
AuNRB: gold nanoraspberries
AuNS: gold nanospheres
