# Supplementary material for: Gold Nanoraspberries for Surface-Enhanced Raman Scattering: Synthesis, Optimization, and Characterization
Source: ACS Omega. 2025 Jan 28;10(5):4588–98. doi: 10.1021/acsomega.4c08791 (PMC11822696; doi:10.1021/acsomega.4c08791)
Supplement: Supplementary file 1 — ao4c08791_si_001.pdf [file ao4c08791_si_001.pdf]

SUPPLEMENTARY INFORMATION

Gold Nanoraspberries for Surface-Enhanced Raman Scattering: Synthesis, Optimization, and Characterization

*Megha Mehta [a], William Skinner [a¬], Benjamin Gardner [a], Sara Mosca [b], Francesca Palombo [a], Pavel Matousek [b], and Nick Stone [*a]*

[a] Department of Physics and Astronomy, University of Exeter, Exeter EX4 4QL, UK

[b] Central Laser Facility, Research Complex at Harwell, STFC Rutherford Appleton Laboratory, UKRI, Harwell Campus, Oxfordshire, OX11 0QX, United Kingdom

[*a] Corresponding author: N.Stone@exeter.ac.uk

AuNRB **AuNS**

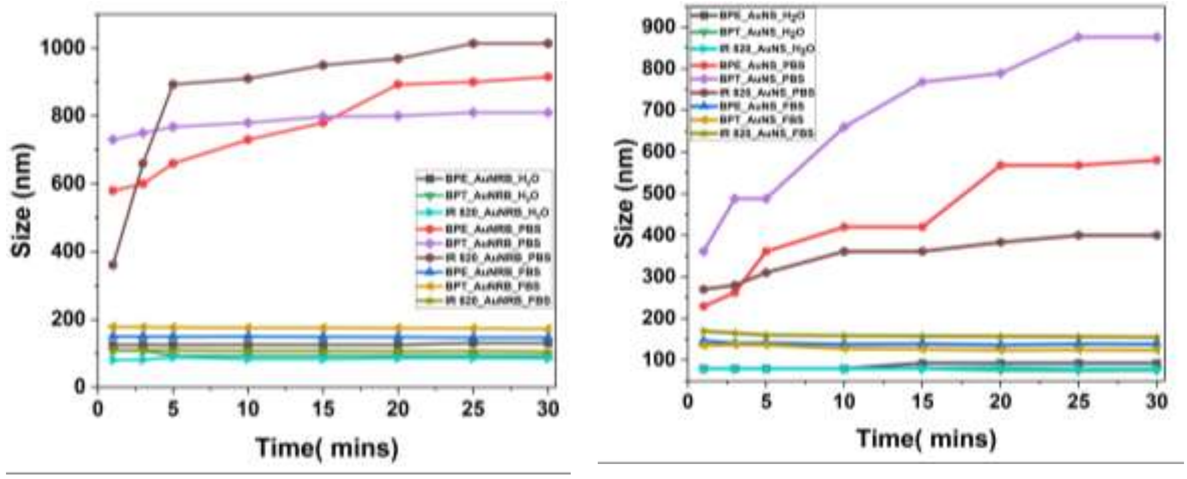

Figure S1. DLS size measurements of AuNRB and AuNS tagged with 5μM BPE, BPT, and IR 820 and later dispersed in ultrapure water, PBS, and 10% FBS at different time intervals to determine the stability of nanoclusters.

AuNRB in water at different magnification

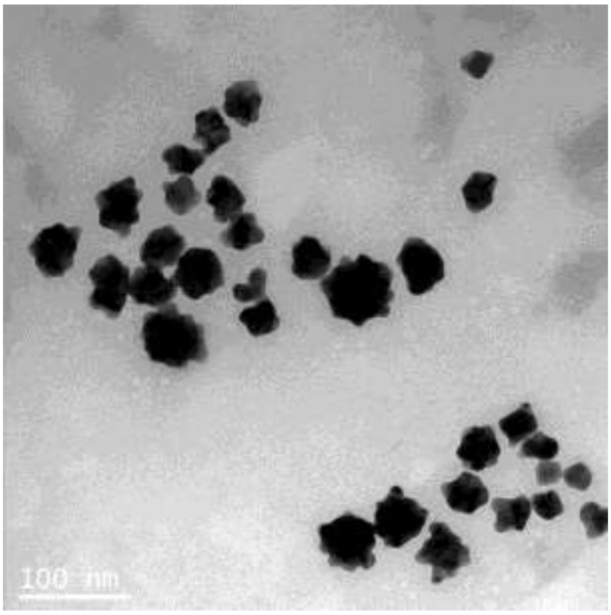

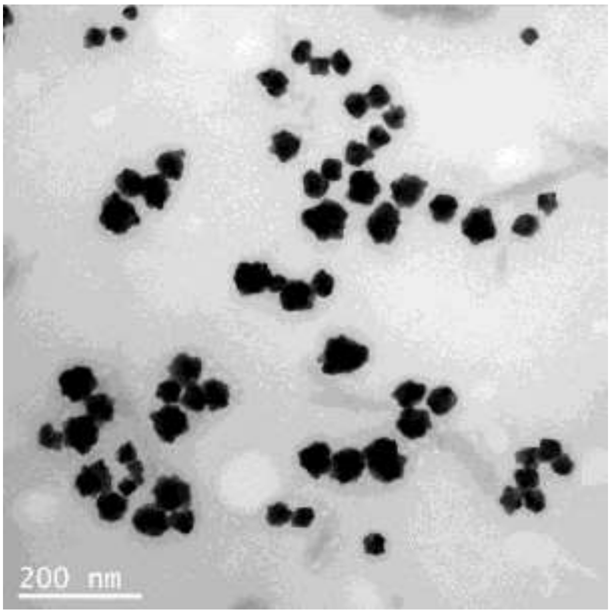

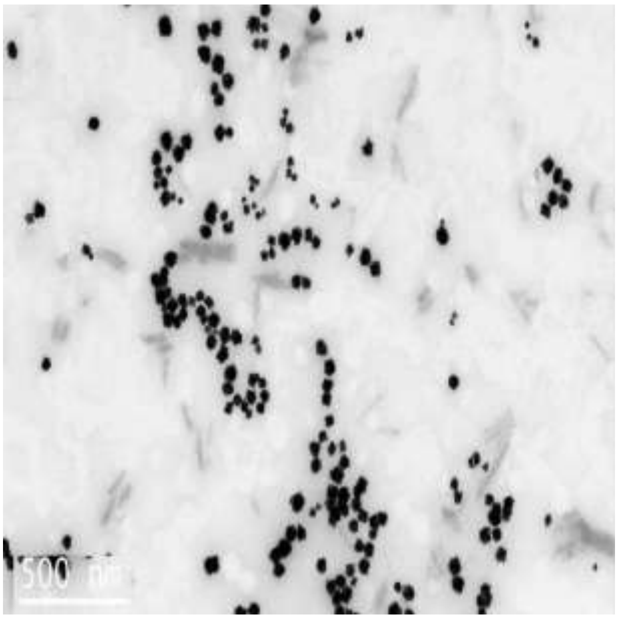

<u>AuNRB in PBS at different magnification</u>

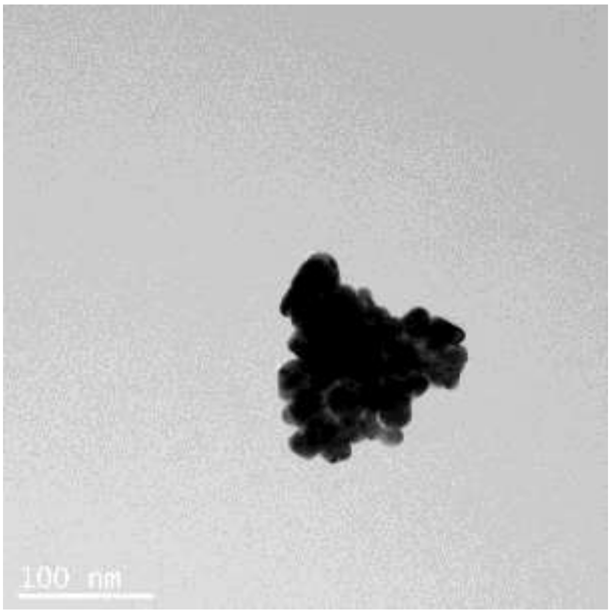
100 nm

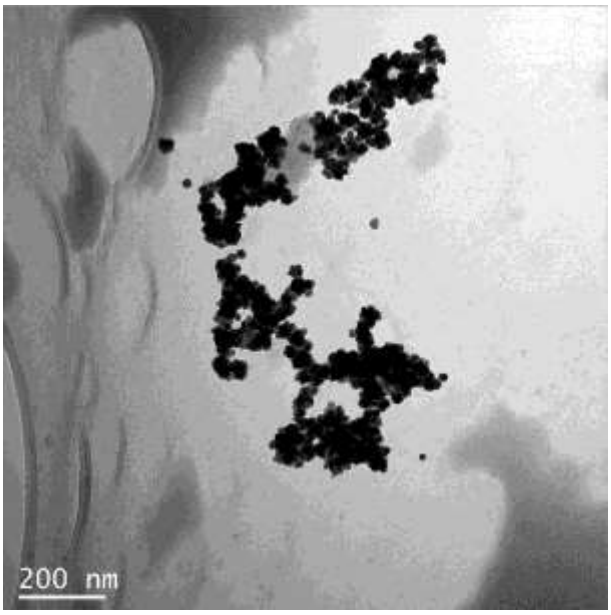
200 nm

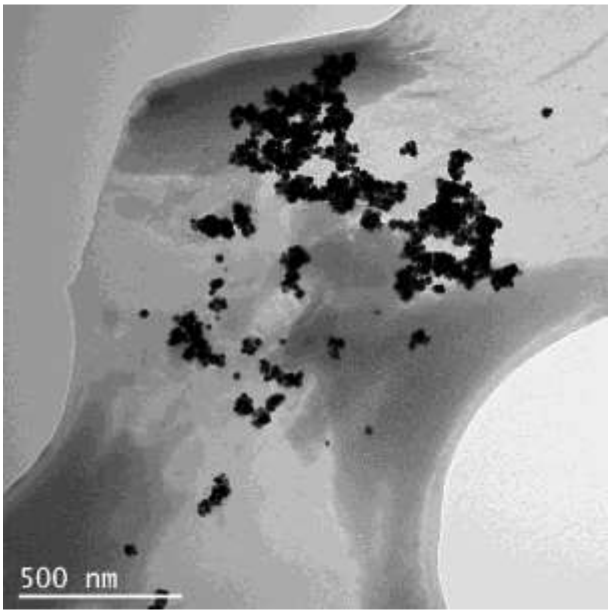

AuNRB in 10% FBS at different magnification

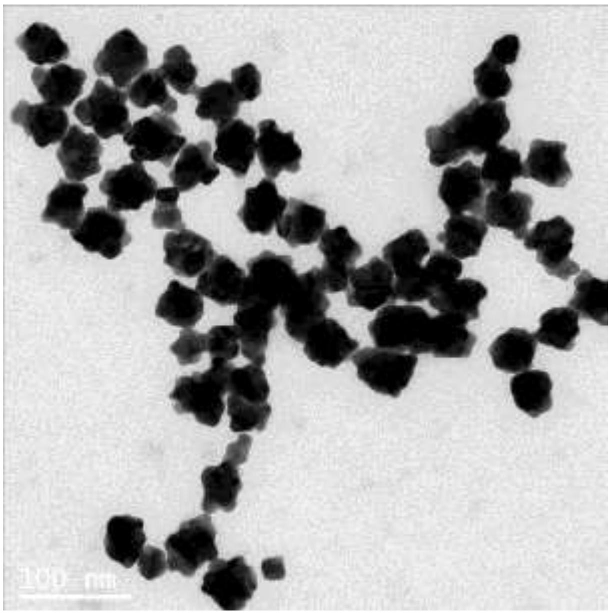

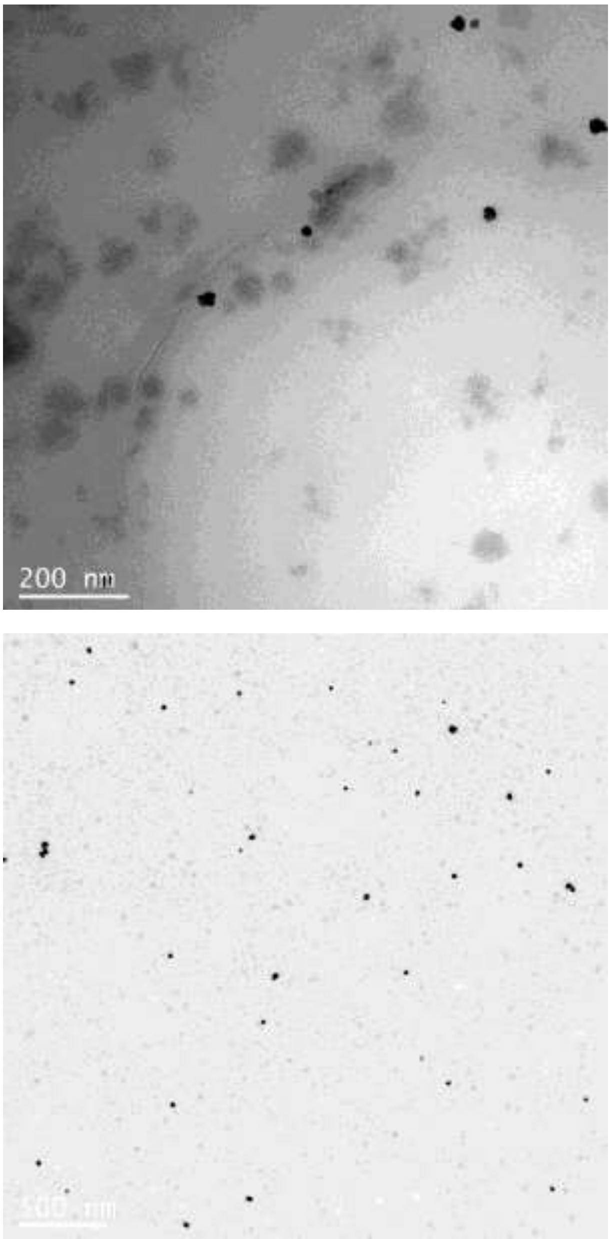

Figure S2. TEM measurements of AuNRB dispersed in ultrapure water, PBS, and 10% FBS at different magnification (100, 200, and 500 nm) levels for better structural characterisation.

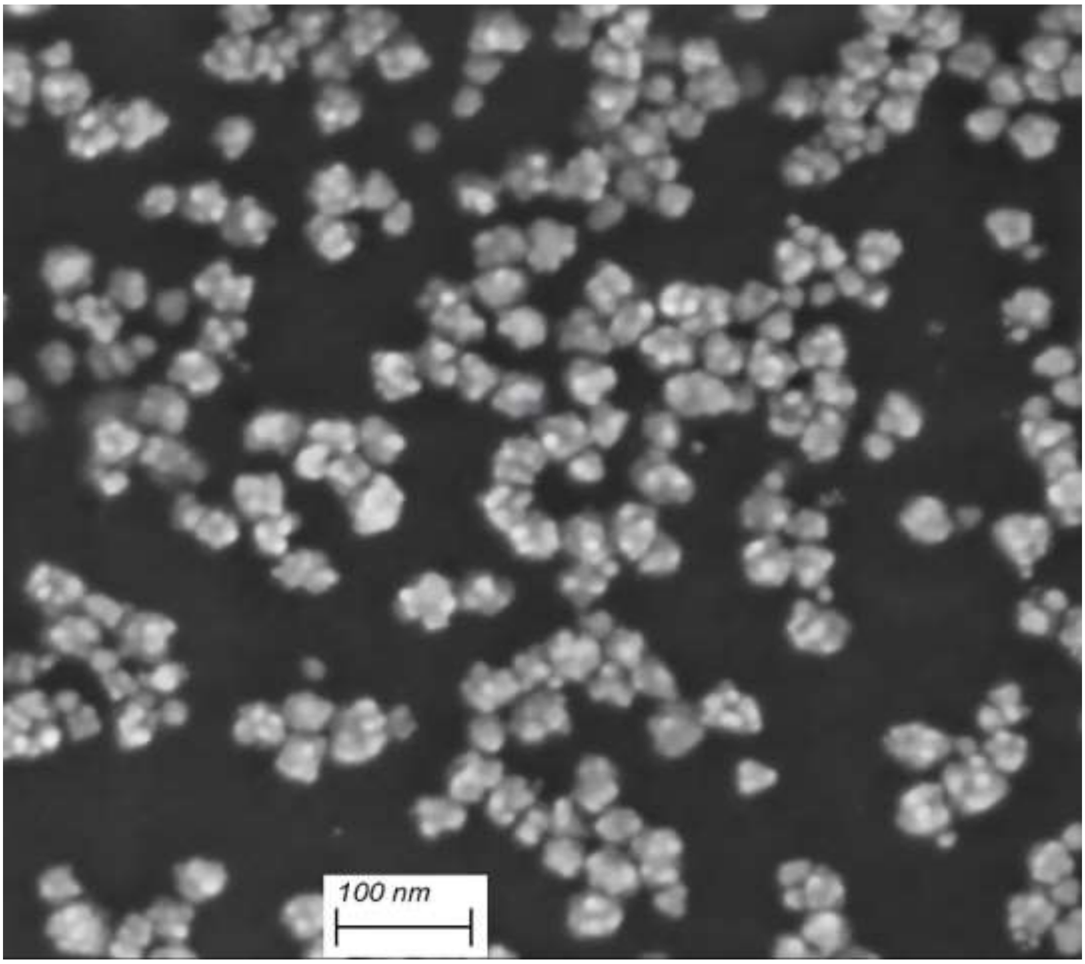

Figure S3. A representative SEM image of Au nanoraspberries disperse in ultra-pure water.

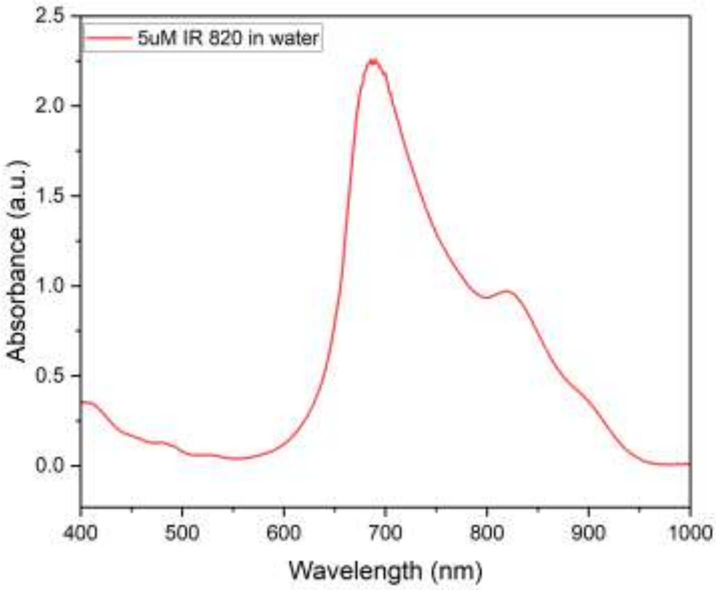

Figure S4. UV-Vis absorbance of 5 μM IR 820 solution.

1. Enhancement Factor

To evaluate the enhancement factor (EF), we compared the difference between the SERS intensities to that of the non-enhanced Raman scattering (normal) using the following expression:

$$EF = \frac{I_{SERS} / C_{SERS}}{I_{Raman} / C_{Raman}} \quad \text{...}(1)$$

where I_{SERS} and I_{Raman} are, respectively, the SERS and normal Raman intensities of reporter molecule and C_{SERS} and C_{Raman} are, the SERS and normal Raman concentration of reporter molecule.

The Raman mode of BPT at 1588 cm^{-1}, BPE at 1608 cm^{-1} and IR-820 at 1360 cm^{-1} were chosen for the EF calculations.

For instance, The SERS signal intensity of 5 μM BPE dispersed in 1 mL of ultrapure water at 1588 cm^{-1} is 1.94335×10^{4} counts and normal Raman signal intensity of 1mM BPE at 1588 cm^{-1} is 4800 counts. We calculated EF using the expression (1) and obtained the value of 3.11×10^{4}. Similarly, we have calculated the EF of BPT and IR-820 labelled gold nanoraspberries 8.63×10^{4} and 1.64×10^{6}.
